# The effect of implicit learning on functional connectivity in schizophrenia

**DOI:** 10.3389/fpsyt.2025.1600449

**Published:** 2025-06-17

**Authors:** Asli Ceren Hinc, Simay Selek, Ibrahim Sungur, Kaan Keskin, Furkan Yazici, Mehmet Can Erata, Yigit Erdogan, Alpaslan Yilmaz, Omer Kitis, Mehmet Cagdas Eker, Ali Saffet Gonul

**Affiliations:** ^1^ Standardization of Computational Anatomy Techniques for Cognitive and Behavioral Sciences (SoCAT) Lab, Department of Psychiatry, School of Medicine, Ege University, Izmir, Türkiye; ^2^ Department of Psychiatry, Izmir City Hospital, Izmir, Türkiye; ^3^ Department of Psychiatry, Ege University, Izmir, Türkiye; ^4^ Department of Child and Adolescent Psychiatry, University of Health Sciences, Erenkoy Mental and Neurological Diseases Research, Istanbul, Türkiye; ^5^ Faculty of Sport Sciences, Erciyes University, Kayseri, Türkiye; ^6^ Department of Radiology, Ege University, Izmir, Türkiye; ^7^ Department of Psychiatry and Behavioral Sciences, Mercer University, School of Medicine, Mocon, GA, United States

**Keywords:** schizophrenia, neuroplasticity, serial reaction time task, fMRI, implicit learning, neuroimaging, resting state, motor learning

## Abstract

**Introduction:**

Neuronal plasticity, or the ability to change and adapt in response to experiences, learning, or environment, is frequently disrupted in schizophrenia and contributes to disease-associated cognitive deficits and functional impairments.

**Methods:**

In this study, we investigated the neuroplasticity alterations of schizophrenia patients in the cortico-striato-cerebellar circuits associated with implicit learning using a reward-enhanced Serial Reaction Time Task (SRTT) by resting-state functional MRI (rs-fMRI). Forty-two schizophrenia patients and 25 healthy controls underwent pre- and post-task rs-fMRI to evaluate changes in functional connectivity.

**Results:**

Behavioral results indicated that all participants demonstrated shorter reaction times during sequential blocks, schizophrenia patients exhibited lower accuracy suggesting diminished implicit learning. Schizophrenia patients exhibited increased connectivity across cortico-striatocerebellar circuits, which became even more robust and widespread following task completion. Despite impaired performance, this post-task hyperconnectivity may reflect a compensatory mechanism attempting to recruit additional neural resources—albeit in a dysfunctional or inefficient manner. Data-driven analyses confirmed the post-task differences between groups, identifying task-induced connectivity changes in thalamo-cortico-cerebellar circuits as the strongest predictors of a group membership.

**Discussion:**

These findings underscore the role of neuroplasticity impairments in schizophrenia-related cognitive deficits, highlighting potential neural markers for clinical differentiation and paving the way for targeted interventions.

## 1 Introduction

Schizophrenia is a mental disorder affecting approximately 1% of the global population, leading to significant functional impairments (1). Accumulated evidence suggests that cognitive symptoms are the mainstay factor for functional impairments in patients. Difficulties in acquiring, encoding, and retrieving new information—along with challenges in integrating previously learned material—are common cognitive impairments that significantly contribute to functional disability (2). It is thought that the cognitive symptoms stem from disrupted neuroplasticity, hindering the brain’s ability to process environmental stimuli essential for daily functioning (3). One major challenge in studying neuroplasticity in the *in vivo* brain is the limited availability of applicable tools. However, resting-state functional MRI (rs-fMRI), which captures changes in the functional coupling between brain regions following learning, has emerged as a promising method for investigating neuroplasticity-related alterations (4).

The Serial Reaction Time Task (SRTT) is commonly used to assess implicit learning—the unconscious acquisition of skills or knowledge—which is often impaired in individuals with schizophrenia. Reaction times (RTs) typically decrease as they become familiar with the predetermined sequence of cues but show a rebound when random stimuli are introduced. (5–7). The subject learns the predetermined sequence implicitly during the task, so, neuroplasticity is expected to occur in related brain regions. Neuroimaging studies revealed that SRTT primarily engages the motor cortex, premotor cortex (PMC), parietal cortex, basal ganglia, and medial temporal lobe (8–18). Indeed, theoretical models and experimental findings highlight the roles of parallel cortico-cerebellar and cortico-striatal circuits in procedural implicit learning (19–21). In the early stages of learning, the cerebellum facilitates motor adaptation and rapid skill acquisition, while the striatum plays a more prominent role in consolidating learned skills over time. Functional connectivity studies suggest a shift in connectivity during learning, decreased cerebellar and increased striatal connectivity to the motor cortex, reflecting the transition from adaptation to consolidation (19).

In the current study, we incorporated a reward-enhanced SRTT protocol between two rs-fMRI sessions to assess neuroplasticity deficits in the cortico-striato-cerebellar circuit and evaluate differences between schizophrenia patients and healthy controls. Based on the literature, we propose that the altered neuroplasticity in schizophrenia patients compared to healthy controls can be assessed by observing functional connectivity changes after SRTT. We included a reward component in SRTT to investigate its modulatory effect on learning and neuroplasticity, given that it has been shown to modulate both implicit and explicit learning (22) and enhance striatal-prefrontal connectivity in motor tasks (23). The use of a reward-enhanced SRTT may help to alleviate motivational deficits associated with negative symptoms and, in turn, enhance cortico-striatal activation during task performance. Thus, this study also highlights the potential role of motivation and reward mechanisms in mitigating these deficits.

## 2 Materials and methods

### 2.1 Participants

A sample of 44 participants with a DSM-5 diagnosis of schizophrenia (age; mean + SD: 35.23 ± 7.77) from the Department of Psychiatry, Ege University School of Medicine, and 26 right-handed healthy controls (age; mean + SD: 36.35 ± 8.04) with similar age, sex distribution, and education were enrolled in this study. The inclusion criteria were: 1) between 18 and 45 years, 2) diagnosis of schizophrenia for at least one year, and have no comorbid psychiatric diagnoses, including substance or alcohol use disorder, and no past present or past psychiatric diagnosis for the controls; 3) for patients, currently in remission according to Andreasen criteria (24) and clinically stable for the past three months (i.e. no changes in symptoms severity requiring interventions such as medication adjustments or hospitalization) 4) completing at least 8 years of basic primary school education 5) using the right hand. The exclusion criteria for both patient and control groups were: 1) unstable chronic or systemic medical diseases; 2) a history of loss of consciousness lasting longer than 3 minutes; 3) the presence of any lesion or space-occupying mass in the MRI; 4) any condition that prevents scanning in a magnetic field (such as having a pacemaker, prosthetics, pregnancy, or claustrophobia).

Participants were enrolled in the study between July 2020 and February 2023. The study was approved by the Ethics Committee of Ege University (approval number 19-12T/42, approval date December 11, 2019) and conducted following the principles of the Declaration of Helsinki from September 2020 to July 2024. After explaining the aim of the study, informed consent was obtained from the patients and their relatives.

### 2.2 Clinical assessments

Trained psychiatrists (ACH, FY) conducted interviews with all participants using the Structured Clinical Interview for DSM 5 (SCID) to confirm diagnoses. Patients were assessed with the following scales: the Positive and Negative Syndrome Scale (PANSS), the Calgary Depression Scale (CGS), the Extrapyramidal Symptom Rating Scale (ESRS), the Edinburgh Handedness Inventory (EHI) to confirm right-handedness, and the Montreal Cognitive Assessment (MoCA) within the week of MRI scans.

### 2.3 Experimental design and procedure

**Figure 1:**
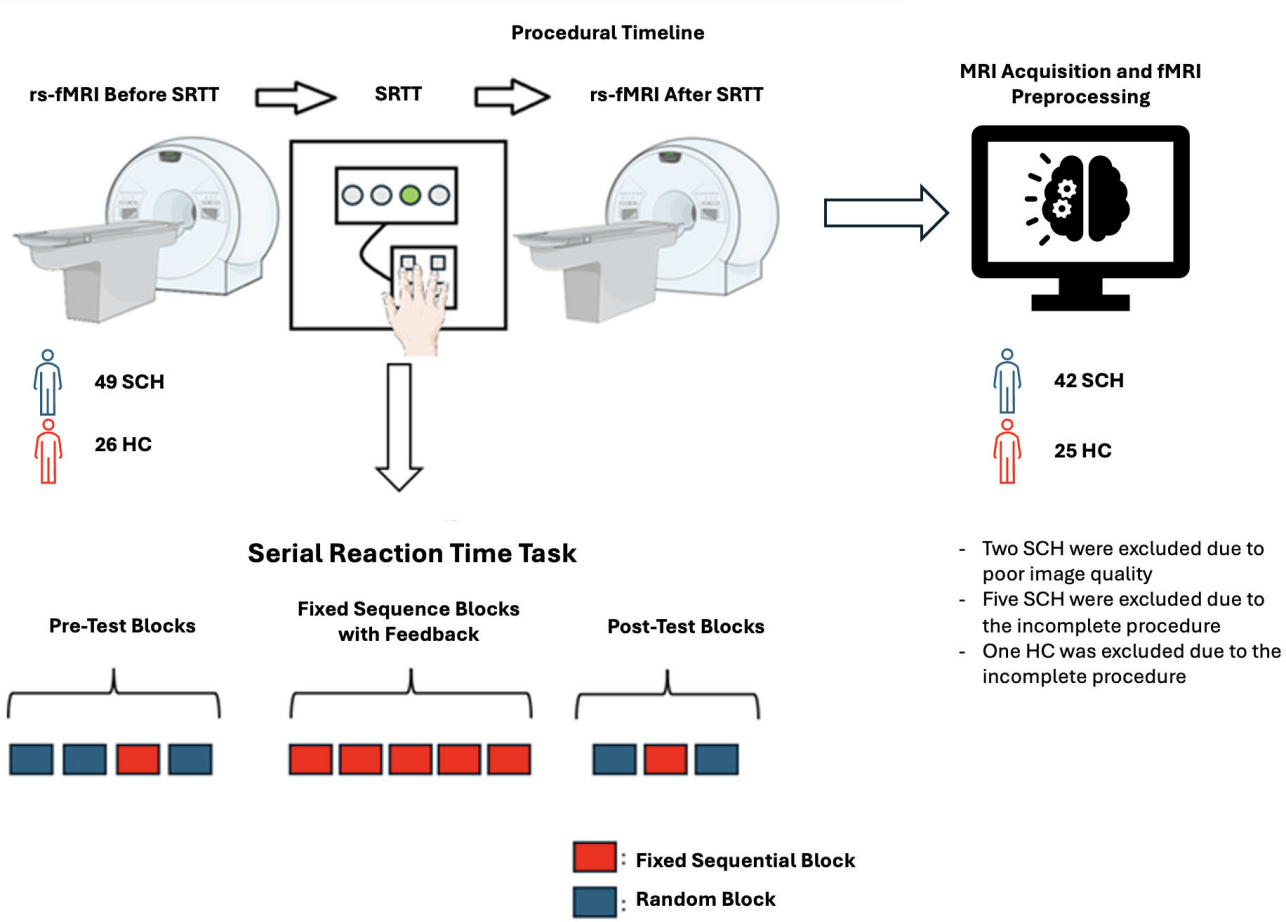
Experiment/Procedure Timeline (Figures contain modified Images from Servier Medical Art (https://smart.servier.com) licensed by a Creative Commons Attribution 3.0 Unported License.

Two resting-state fMRI scans were conducted on participants—one before and one after the Serial Reaction Time Task (SRTT). (**Figure 1**).

#### 2.3.1 Serial reaction time task

The Serial Reaction Time Task (SRTT) was developed in-house using the Borland Delphi software version 7. In this task, a visual cue appears at one of four horizontally arranged positions on a computer screen, labeled 1 through 4, corresponding to specific keys on the keyboard (‘g,’ ‘h,’ ‘j,’ ‘k’). At the onset of each trial, when the stimulus is presented (i.e., the gray box turns white), the participant must press the appropriate key using the designated fingers. A correct response within 800 ms causes the box to turn green, while a late or incorrect response triggers a red “error” message, which is displayed for 100 ms. The reaction time (RT), defined as the time taken to press the correct key, is recorded for each trial. Thus, reaction time, which is the primary performance measure, cannot exceed 800 ms.

The task consists of 12 blocks. Each block contains 96 trials and is separated by a 3000 ms rest period. Initially, the visual stimuli are presented randomly, followed by a fixed sequential order (e.g., 3-4-1-2-3-1-4-3-2-4-2-1) for implicit learning. Afterward, the sequence is randomized again, and the participant’s RT is recorded for each trial (4).

The first two blocks serve as “practice random blocks,” where stimuli are presented randomly, ensuring no stimulus appears more than twice consecutively. The third block introduces the “fixed sequential sequence” (e.g., 3-4-1-2-3-1-4-3-2-4-2-1), which is repeated eight times within the block. This block is referred to as the “pre-test fixed sequential block”. The fourth block returns to random stimuli. Blocks five through nine consist of the fixed sequential sequence, but with feedback provided during the 3000 ms rest periods between blocks. These blocks are referred to as the “fixed sequential blocks with feedback”. Feedback is contingent on the participant’s performance: if the error rate in a block is below 5%, the feedback message “you won money” is displayed; otherwise, “try harder!” is shown.

The tenth and twelfth blocks consist of random stimuli, while the eleventh block is another fixed sequential block without feedback. This block is referred to as the “post-test fixed sequential block”. Participants are informed prior to the task that they will earn a monetary reward; with the amount they can earn determined by their performance. After completing the task, participants were asked whether they could identify the presence of the sequence and, if they could, recall the sequence (25). Subsequently, all participants received a monetary reward of $10.

### 2.4 MRI acquisition and fMRI preprocessing

MRI Acquisition and fMRI preprocessing details are given in **Supplementary Material** with a standard minimal preprocessing driven by CONN’s default pipeline (26).

### 2.5 Image and statistical analysis

#### 2.5.1 fMRI data analyses

Before and after the given task (SRTT), rs-fMRI was conducted to detect brain functional changes resulting from implicit learning in the schizophrenia group compared to the healthy control group. This analysis was performed using a 2x2 ANOVA generalized linear model (GLM) with the Functional Connectivity Toolbox (CONN Toolbox/(RRID: SCR_009550) release 22.a) based on MATLAB/Statistical Parametric Mapping (SPM/(RRID: SCR_007037) release 12.7771). Outliers were identified and removed through the CONN, yielding a final sample of 45 schizophrenia patients and 22 healthy controls.

**Figure 2:**
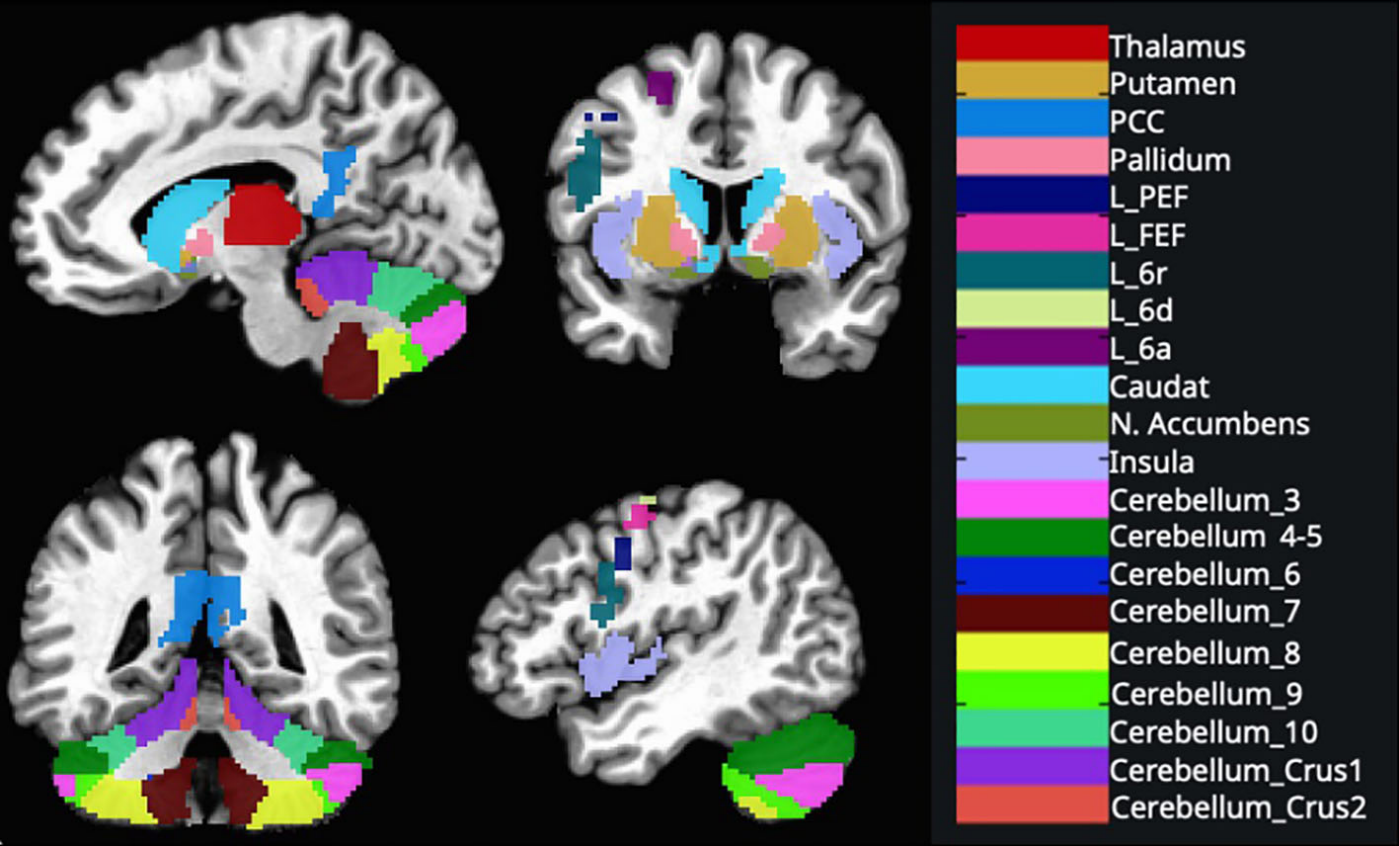
These regions were created using the AAL 3v1 atlas and overlaid on the MNI152 template brain and AFNI’s 3dcalc tool. (L, Left; FEF, frontal eye field; PCC, posterior cingulate cortex; PEF, posterior eye field) See **Supplementary Material** for ROI coordinates.

Based on the previous studies about motor learning and executive tasks, we implemented ROI-to-ROI analysis of the posterior cingulate cortex (PCC), insular cortex (IC), thalamus, caudate nucleus, putamen, pallidum, nucleus accumbens, cerebellum’s subdivisions bilaterally and the left premotor area’s subdivisions (27) (**Figure 2**).

Functional and anatomical data were preprocessed using a modular preprocessing pipeline, which included realignment with correction of susceptibility distortion interactions, slice timing correction, outlier detection, direct segmentation and MNI-space normalization, and smoothing.

After preprocessing, data from 67 participants were used to generate seed-based connectivity maps (SBC) and estimate ROI-to-ROI connectivity matrices (RRC), characterizing functional connectivity patterns across 183 ROIs. In addition to the CONN toolbox default ROI parcellation, we prepared ROIs from glasser parcellation to investigate the premotor region more deeply.

The Glasser HCP atlas (MNI_Glasser_HCP_v1.0_LPI_2009c_resampled.nii.gz) in AFNI was used to distinguish the superior and inferior premotor subdivisions in the bilateral hemispheres. According to the Glasser atlas, the superior premotor subdivisions include 6d, 6a, and the frontal eye field (FEF), while the inferior premotor subdivisions include 6v, 6r, and the premotor eye field (PEF). These subdivisions were extracted for both the left and right hemispheres. Binary masks for each premotor region of interest (ROI) were created using AFNI’s 3dcalc tool.

Functional connectivity strength was represented by Fisher-transformed bivariate correlation coefficients computed between each pair of seed and target ROIs, modeling the association between their BOLD signal time series. For each condition (pre-task and post-task), connectivity was estimated using the default weighted correlation measures provided by the CONN toolbox. Group-level analyses were performed using a General Linear Model (GLM). Separate between-group comparisons (schizophrenia vs. control) were conducted for the pre-task and post-task resting-state conditions. For each ROI pair, connectivity values served as the dependent variable, with group as the independent variable. Inferences were assessed using cluster-level statistics across ROI-to-ROI connections based on parametric multivariate statistics (28), with a cluster-level false discovery rate (p-FDR < 0.05) threshold and connection-level p < 0.05 uncorrected threshold.

#### 2.5.2 Behavioral data analyses

Behavioral data recorded during the SRTT were analyzed using a mixed-design variance analysis: 2 (group: control and schizophrenia) x 2 (sequence type: random and sequential) x 2 (test time: pre-test and post-test) to investigate whether there were differences in mean RT and the number of correct responses between groups in pre-test and post-test sequential and random blocks. Bonferroni correction was applied for Tukey’s *post-hoc* analyses. All results were considered significant at an alpha level of 0.05, and effect sizes were reported as partial eta squared.

To assess the changes in average RT and the number of correct responses during the five blocks of the fixed sequential sequence between the groups, each block was included in a 2 (group: control and schizophrenia) x 5 (block 5-6-7-8-9) ANOVA. Bonferroni correction was also applied for *post-hoc* analyses. Again, all results were considered significant at an alpha level of 0.05, and effect sizes were reported as partial eta squared. A correlation analysis was performed between the behavioral data and brain imaging results of the two groups.

We used the Shapiro-Wilk test was used to assess normality. Independent sample t-tests were conducted to compare the age differences, Pearson Chi-Square for gender differences, and Mann Whitney U for education year differences between patients and controls. We conducted a correlation analysis between age-task performance, clinical characteristics-functional connectivity, and behavioral data-functional connectivity with Pearson and Spearman correlation, respectively.

#### 2.5.3 Classification method

Here, our primary aim was to investigate how neural alterations and behavioral outcomes after task completion contribute to the classification of subjects. Specifically, we analyzed “after > before” and “schizophrenia > control” contrasts using regions identified with p <.05, uncorrected, in CONN. We included both conditions separately without performing subtraction to evaluate whether post-task measurements were better predictors than pre-task measurements.

Subsequently, we applied a False Discovery Rate (FDR) correction and selected significant ROIs for further analysis. We included mean reaction time and accuracy rate as additional variables as part of a control analysis. Given the high multicollinearity among predictors, we employed Principal Component Analysis (PCA) with scaling to prepare the final predictor matrix.

For classification, we utilized the Support Vector Machine (SVM) approach. To classify the data, we implemented a Support Vector Machine (SVM) with a radial basis function (RBF) kernel using the caret package in R. Hyperparameter optimization was performed to tune the kernel width (σ) and the regularization parameter (C). A 10-fold cross-validation procedure repeated 10 times (i.e., 10x10 repeated cross-validation) was employed to ensure robust model evaluation and to mitigate overfitting. The tuning process involved a grid search over the σ∈ {0.01, 0.1, 0.5, 1} and C ∈ {0.01, 0.1, 1, 10, 100}.

Model performance during tuning was evaluated using the area under the receiver operating characteristic curve (AUC-ROC) as the metric. The optimal hyperparameters (sigma = 0.01 and C = 0.01) were selected based on this criterion. Subsequently, the dataset was randomly partitioned into training (80%) and testing (20%) sets using stratified sampling. The final SVM model was retrained on the training set using the optimal hyperparameters and evaluated on the testing set. After training and testing the model, we conducted a permutation analysis on the principal components to assess their relationship with clinical classification.

To evaluate the contribution of each principal component (PC) to the SVM classifier’s performance, we conducted a permutation-based feature importance analysis on the test set. This procedure quantifies how much the model’s predictive ability deteriorates when the values of a given PC are randomly permuted, thereby disrupting its relationship with the target variable.

Specifically, for each principal component, the values of the component in the test set were independently permuted while keeping all other components unchanged. Afterwards, predictions were generated using the trained SVM model on the permuted test data. The area under the receiver operating characteristic curve (AUC-ROC) was calculated based on those predictions. Lastly, the AUC drop was computed by subtracting the AUC of the permuted data from the original (unpermuted) AUC, representing the importance of that component. This process was repeated for each PC, and the resulting AUC drops were used to rank the components by their contribution to the model’s performance.

Finally, to enhance generalizability and reduce bias, we included all functional connectivity (FC) between regions—both pre-task and post-task—as predictors in the SVM model. This approach ensured that the classification was not limited to features that specifically differentiated the schizophrenia (SZ) and healthy control (HC) groups, thereby minimizing the risk of overfitting to group-specific patterns.

## 3 Results

### 3.1 Demographic and clinical characteristics

After excluding eight participants (two due to poor image quality and six for incomplete procedural data), the final sample consisted of 67 participants, with 42 patients and 25 controls. The two groups were similar in age, gender, and education (**Table 1**).

**Table 1:** Demographic and clinical characteristics of schizophrenia and control groups.

Variable	Schizophrenia (N = 42)	Controls (N = 25)	Statistics
Age, mean years (SD)	35.4 (7.7)	36.3 (8.2)	*t* = 0.57 df= 65 *P=* 0.66
Female (%)	17 (40.5%)	13 (52%)	*X* ^2^ = 0.841 df= 1 *P=* 0.35
Education, median years (SD)	12.9 (3.0)	12.4 (3.5)	*t* = 0.65 df = 44 *P=* 0.62
Onset, mean years (SD)	21.9 (5.4)	NA	NA
Duration, mean years (SD)	13.5 (7.0)	NA	NA
PANSS, mean (SD)	63.2 (12.4)	NA	NA
P-PANSS, mean (SD)	10.7 (3.3)	NA	NA
N-PANSS, mean (SD)	21.8 (7.4)	NA	NA
G-PANSS, mean (SD)	30.6 (5.5)	NA	NA
SANS, mean (SD)	52.4 (418.8)	NA	NA
CDS, mean (SD)	2.4 (4.2)	NA	NA
ESRS, mean (SD)	5.6 (5.6)	NA	NA
CPZ, mean (SD)	583.4 (369.0)	NA	NA
Antipsychotic type		NA	NA
FGAs (%) SGAs (%) FGAs+SGAs (%)	0 (0%) 36 (87.8%) 5 (12.2%)	NA NA NA	NA

CDS, Calgary Depression Scale; CPZ, Chlorpromazine equivalent doses; ESRS, Extrapyramidal Symptoms Rating Scale; FGAs, first-generation antipsychotics; G-PANSS, Positive and Negative Syndrome Scale, general symptoms subscale; N, number of individuals; N-PANSS, Positive and Negative Syndrome Scale, negative symptoms subscale; NA, not available; PANSS, Positive and Negative Syndrome Scale; P-PANSS, Positive and Negative Syndrome Scale, positive symptoms subscale; SANS, Scale for the Assessment of Negative Symptoms; SGAs, second-generation antipsychotics; SD, Standard Deviation. Two patients have no PANSS, SANS, CDS, or ESRS score data, and one has no medication information.

### 3.2 Behavioral data

#### 3.2.1 SRTT performance in pre-test and post-test blocks: mean RT and correct responses

**Figure 3:**
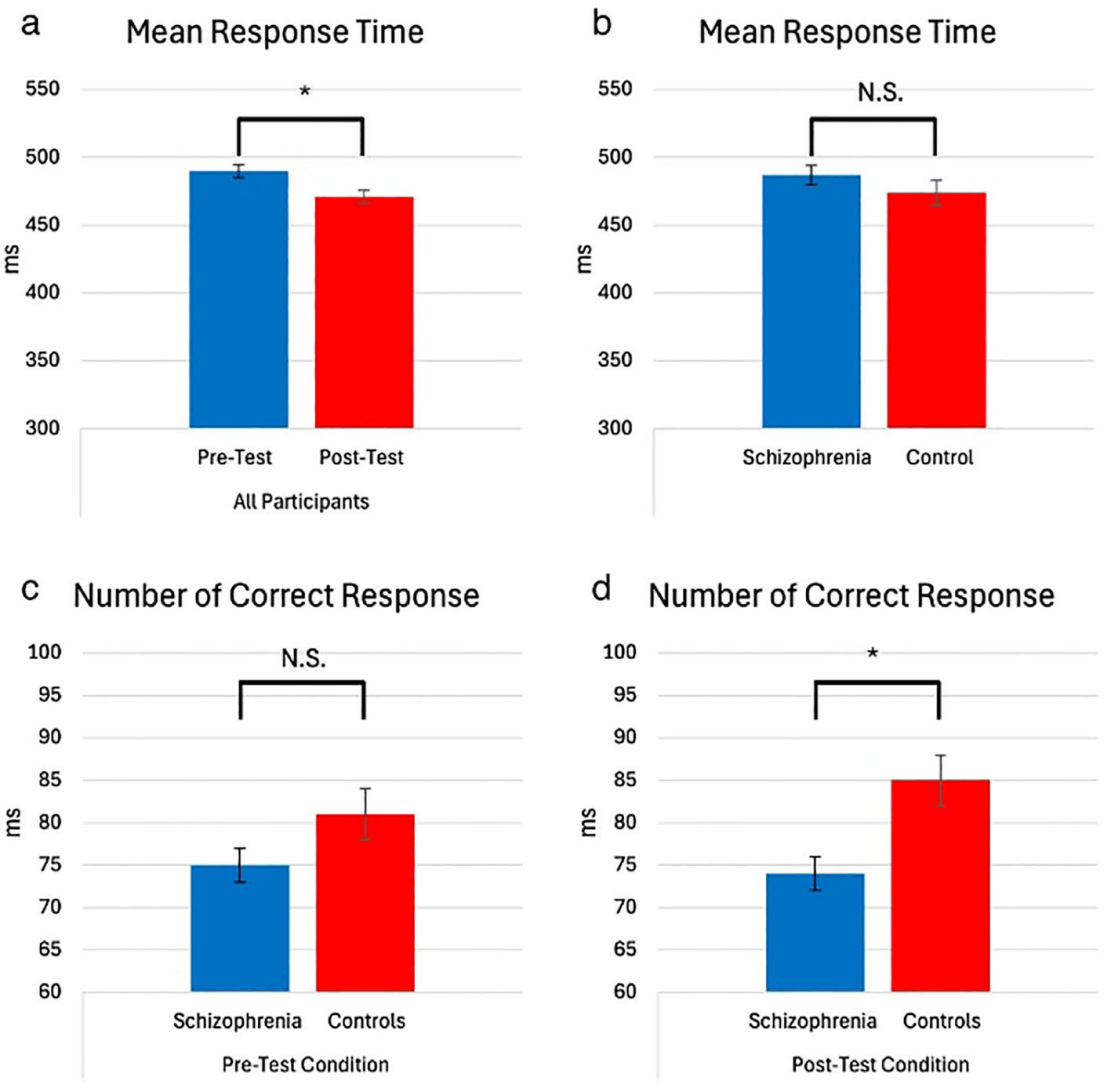
Mean RT (milliseconds) and number of correct responses of participants, test time, and group (with 95% Confidence Interval). (* = p<0.05) The main effect of test time on mean reaction time (RT). Participants showed significantly shorter RTs in the post-test (M = 471.21, SE = 5.88) compared to the pre-test (M = 490.25, SD = 5.73). No group-related interactions were observed, which mainly affected the number of correct responses. In the pre-test, no significant difference in performance between groups was observed (M_SCZ = 75.53, SE = 2.17 vs. M_HC = 81.64, SE = 2.82), but in the post-test, the control group outperformed the schizophrenia group (M_SCZ = 74.10, SE = 2.39 vs. M_HC = 85.02, SE = 3.10) **(a)** Mean Response Time – All Participants (Pre-Test vs. Post-Test). **(b)** Mean Response Time – Schizophrenia vs. Control. **(c)** Number of Correct Responses – Pre-Test Condition (Schizophrenia vs. Controls). **(d)** Number of Correct Responses – Post-Test Condition (Schizophrenia vs. Controls). NS, not significant.

As expected, all the participants showed better performance for RT during the post-test compared to the pre-test (F(1,65) = 86.49, p < 0.001, η² = 0.57) (**Figure 3a**). There were no group effects among patients and controls, suggesting both groups showed similar performance (F(1,65) = 1.35, p = 0.40, η² = 0.02) (**Figure 3b**). Additionally, all participants showed better performance in the sequential blocks during both the pre-test and post-test (F (1,65) = 11.90, p = 0.003, η² = 0.15). Patients showed poorer performance than controls in the post-test for correct responses (F(1,65) = 7.72, MD = 10.91, SE = 3.92, p = 0.007) (**Figure 3d**), despite no significant difference between the groups in the pre-test (F(1,65) = 2.93, MD = 6.11, SE = 3.56, p = 0.09) (**Figure 3c**). This finding suggests that, compared to controls, patients demonstrated reduced effectiveness in sequence acquisition.

#### 3.2.2 SRTT performance in fixed sequential blocks with feedback: mean RT and correct responses

**Figure 4:**
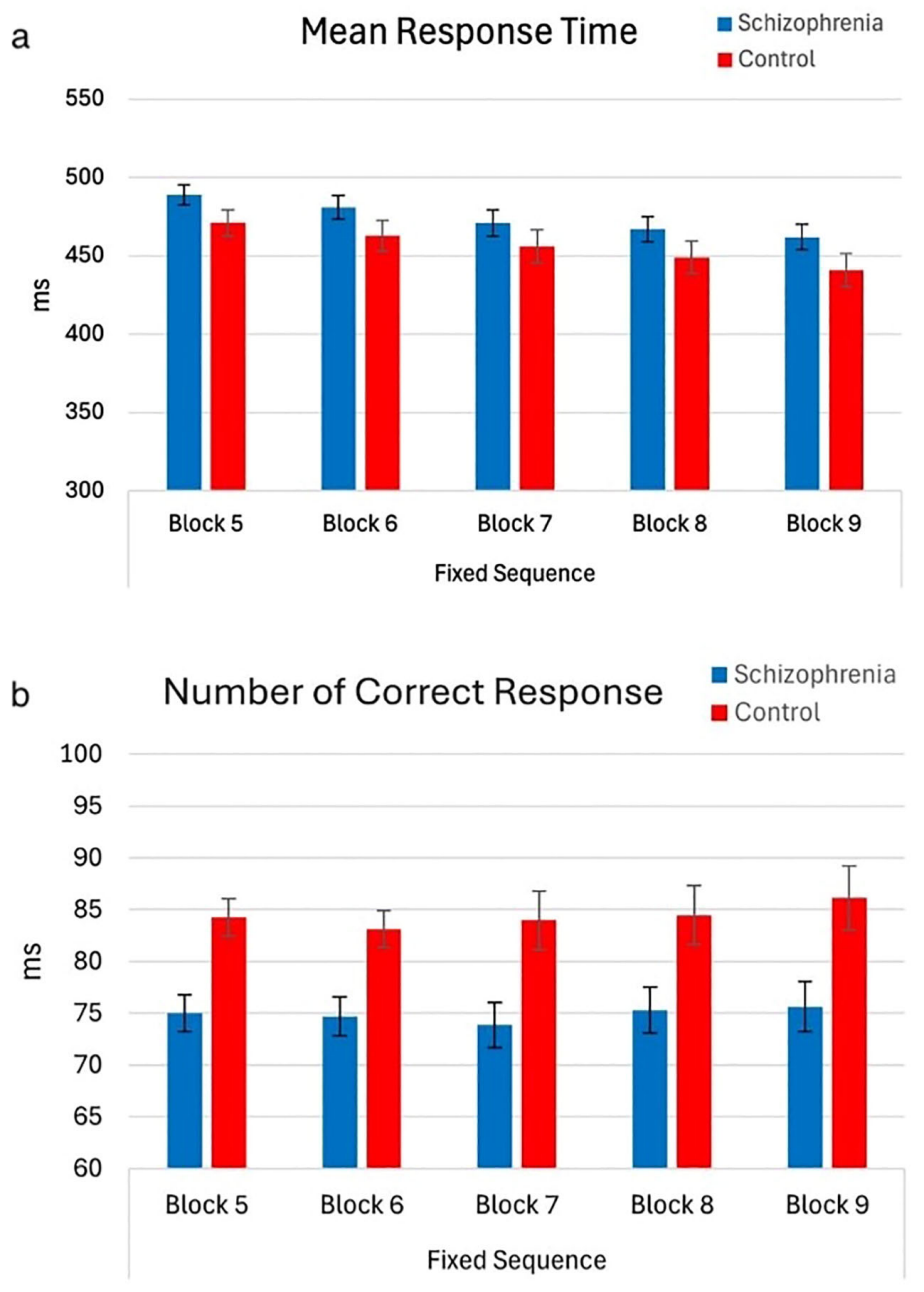
Mean RT and number of correct responses of participants by block order (with 95% Confidence Interval). The main effect of block order on mean reaction time (RT) during the fixed-test period. A significant decrease in mean RT was observed as blocks progressed, with Block 5 (M = 479.86, SE = 5.27) showing slower RTs compared to Block 6 (M = 471.79, SE = 6.17), Block 7 (M = 463.59, SE = 6.71), Block 8 (M = 457.97, SE = 6.51), and Block 9 (M = 451.34, SE = 6.63), indicating a gradual reduction in RTs across blocks. However, the main effect of the group on mean RT was not significant (F(1,65) = 2.44, p = 0.12), nor was the interaction between block order and group (F(1,65) = 0.13, p = 0.81). The main effect of the group on the number of correct responses during the fixed-test period. The number of correct responses in the schizophrenia group (Mean = 74.89, SE = 2.21) is lower than control (Mean = 84.38, SE = 2.86). However, the main effect of block order on the number of correct responses was not significant (F (1,65) = 1.52, p = 0.22), nor was the interaction between block order and group (F (1,65) = 0.38, p = 0.81) **(a)** Mean Response Time – Fixed Sequence (Blocks 5 to 9). **(b)** Number of Correct Responses – Fixed Sequence (Blocks 5 to 9).

As the blocks progressed, both groups responded faster, and the mean reaction times gradually decreased (F (1,65) = 17.45, p < 0.001, η² = 0.21) (**Figure 4a**). There were no group effects on RT (main effect, F (1,65) = 2.44, p = 0.81; group effect, F (1,65) = 0.13, p = 0.95). Nevertheless, regardless of block progression, patients exhibited a lower number of correct responses compared to controls (**Figure 4b**).

### 3.3 Imaging results

**Figure 5:**
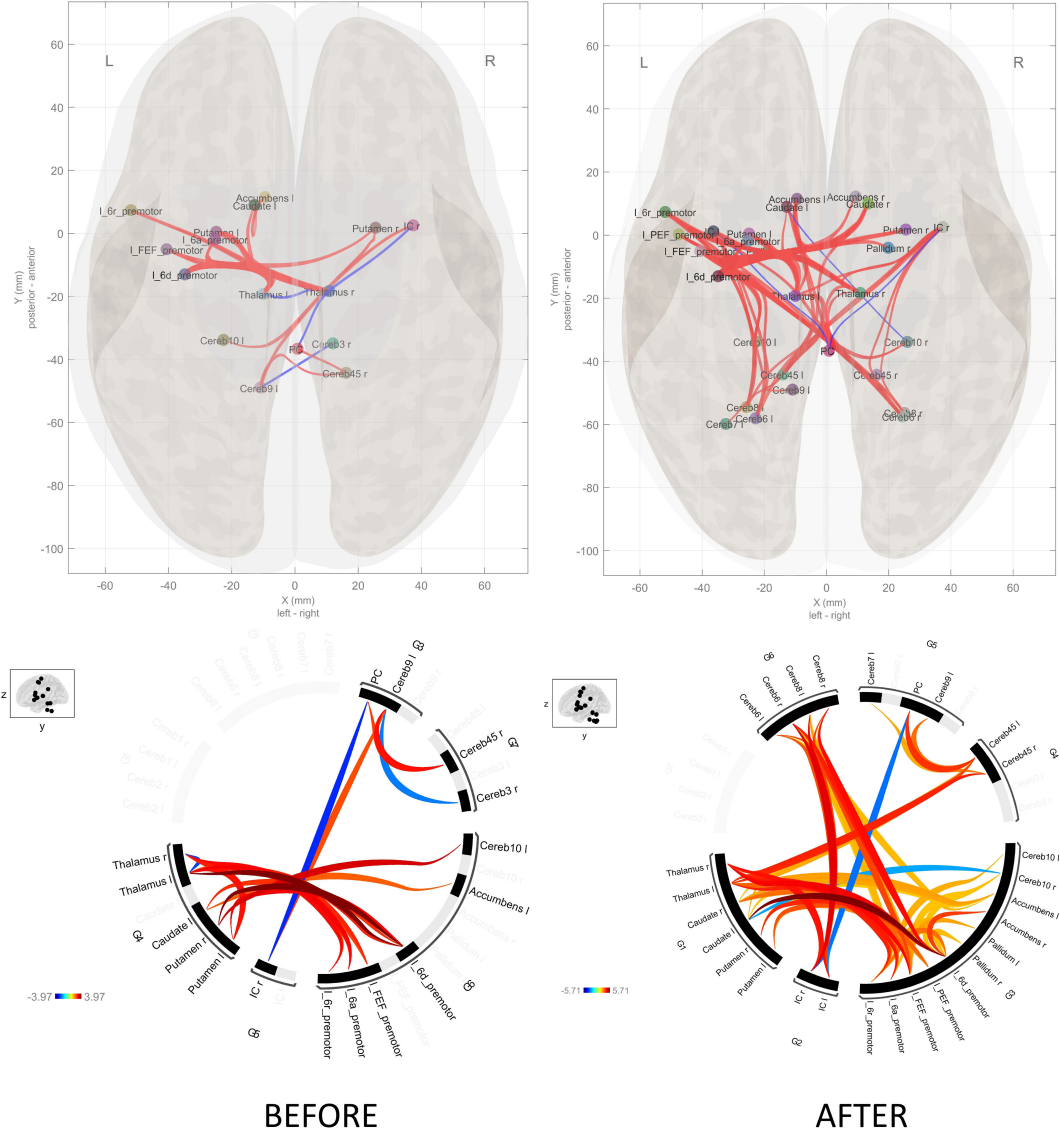
Schizophrenia versus controls connectome diagram before and after the SRTT. The connectome diagram demonstrates the summing of the functional connectivity of the healthy controls from the functional connectivity of the schizophrenia patients. There is an increase in functional connectivity after the SRTT.

In the group comparison at the baseline, schizophrenia patients had higher connectivity between thalamus and putamen with left premotor regions (6a, 6d, FEF) (**Table 2**). Additionally, patients showed higher connectivity between thalamus with putamen and cerebellum (**Table 2**, **Figure 5**). After the task, patients showed increased connectivity between all subdivisions (6a, 6d, 6r, FEF, PEF) of the left premotor cortex (PMC) and the striatum (caudate and putamen), thalamus, cerebellum, and nucleus accumbens (**Table 3**, **Figure 5**). Notably, nucleus accumbens increased connectivity with thalamus. The cerebellum also exhibited increased intra-cerebellar functional connectivity and connectivity with the thalamus and PCC. Insula showed increased connectivity with the thalamus and cerebellum, while decreased connectivity between insula and PCC persisted.

**Table 2:** Schizophrenia versus controls, before the task.

Analysis unit	Statistics	p-FDR
Thalamus_L - 6d_premotor_L	T (65) = 3.97	0.006414
Putamen_R - 6d_premotor_L	T (65) = 3.92	0.007455
Putamen_R - Cerebelum_10_L	T (65) = 3.29	0.019004
Thalamus_L - 6a_premotor_L	T (65) = 3.04	0.028046
Thalamus_L - FEF_premotor_L	T (65) = 2.71	0.042667
Thalamus_L - Putamen_L	T (65) = 2.84	0.034848

L, Left; R, Right; FEF, Frontal Eye Field; FDR: False Discovery Rate).

**Table 3:** Schizophrenia versus controls, after the task.

Analysis unit	Statistics	p-FDR
Caudate_L - 6d_premotor_L	T (65) = 5.71	0.000011
Thalamus_R - 6d_premotor_L	T (65) = 4.38	0.001047
Thalamus_R - FEF_premotor_L	T (65) =4.24	0.001047
Thalamus_R – 6a_premotor_L	T (65) = 4.03	0.001047
Thalamus_R – 6r_premotor_L	T (65) = 3.91	0.001117
Thalamus_L - 6d_premotor_L	T (65) = 3.91	0.004825
Putamen_R - 6d_premotor_L	T (65) = 3.86	0.009184
Thalamus_L - 6a_premotor_L	T (65) = 3.21	0.010221
Thalamus_L - FEF_premotor_L	T (65) = 3.19	0.010221
Thalamus_L - 6r_premotor_L	T (65) = 3.17	0.010221
Putamen_R - PEF_premotor_L	T (65) = 3.19	0.038599
Putamen_L - 6d_premotor_L	T (65) = 3.39	0.042165
Thalamus_R - Accumbens_L	T (65) = 2.48	0.042197
Thalamus_L - PEF_premotor_L	T (65) = 2.54	0.046755
Thalamus_R - Insula_L	T (65) = 4.09	0.001047
Thalamus_R - Insula_R	T (65) = 4.03	0.001047
Thalamus_L - Insula_R	T (65) = 3.70	0.005247
Thalamus_L - Insula_L	T (65) = 3.34	0.009845
6d_premotor_L - Cerebelum_8_R	T (65) = 4.19	0.000990
6d_premotor_L - Cerebelum_6_R	T (65) = 3.96	0.001285
6d_premotor_L - Cerebelum_6_L	T (65) = 3.44	0.003268
FEF_premotor_L - Cerebelum_6_R	T (65) = 3.53	0.013268
Insula_R - Cerebelum_6_R	T (65) = 4.58	0.000527
Insula_R - Cerebelum_8_L	T (65) = 4.49	0.000527
Insula_R - Cerebelum_8_R	T (65) = 3.51	0.004753
Insula_L - Cerebelum_6_R	T (65) = 3.64	0.009516
Insula_L - Cerebelum_6_L	T (65) = 3.39	0.009845
Insula_R - Cerebelum_6_L	T (65) = 3.17	0.011722
Insula_L - Cerebelum_8_L	T (65) = 2.79	0.036146
6d_premotor_L - Accumbens_R	T (65) = 3.60	0.002168
6r_premotor_L - 6d_premotor_L	T (65) = 3.05	0.038478
Thalamus_R - Cerebelum_4_5_R	T (65) = 3.91	0.001117
Thalamus_L - Cerebelum_4_5_R	T (65) = 3.85	0.004825
Thalamus_L - Cerebelum_4_5_L	T (65) = 3.51	0.007238
Thalamus_R - Cerebelum_4_5_L	T (65) = 3.16	0.009368
Insula_R - Posterior Cingulate	T (65) = -3.02	0.015888
Insula_L - Posterior Cingulate	T (65) = -2.69	0.039553
Cerebelum_4_5_L - Posterior Cingulate	T (65) = 3.77	0.012609
Cerebelum_4_5_L - Cerebelum_9_L	T (65) = 3.07	0.022660

L, Left; R, Right; FEF, Frontal Eye Field; PEF, Posterior Eye Field; FDR: False Discovery Rate).

We could not observe any difference between baseline and post-task within the groups.

### 3.4 Classification results

We conducted an analysis to evaluate how effectively task could distinguish between two clinical groups, using a classification method called Support Vector Machine (SVM). We built a model based on data from brain regions of interest (ROIs), including differences in activity before and after task, response times, and number of correct responses (see Method - Classification Method). To fine-tune the model, we optimized its settings to achieve the best performance, measured by an evaluation metric Area Under the Curve (AUC). The model performed well, achieving an overall AUC of 0.95 on train dataset, and a perfect score of 1 on the test dataset.

**Figure 6:**
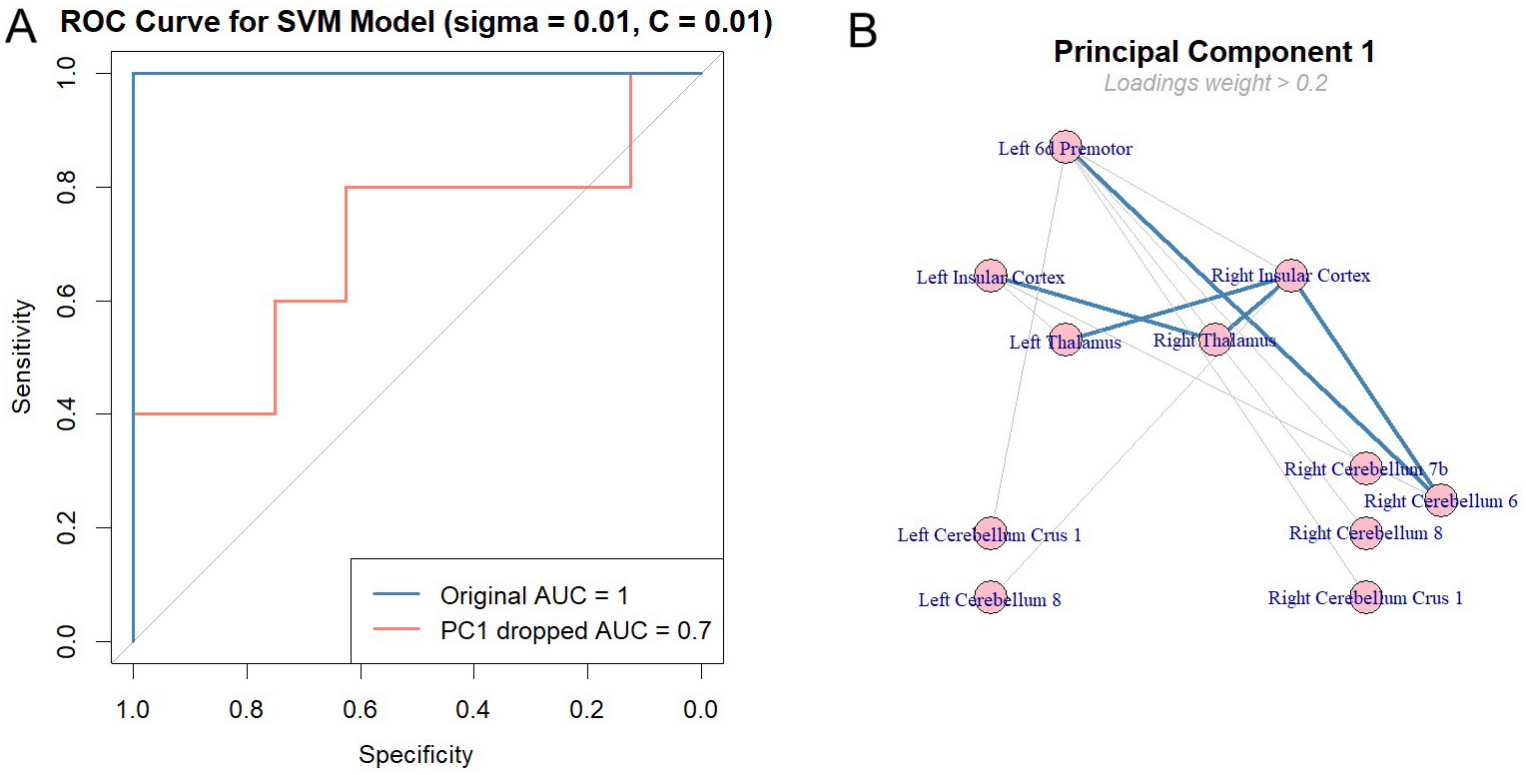
Classification of clinical groups by task effect. **(A)** The Support Vector Machine (SVM) model was trained on 80% of the data using a 10x10 nested cross-validation approach to ensure reliable and robust performance. Hyperparameters, specifically sigma and C, were optimized through a grid search, with both values fixed at 0.01 as they yielded the best performance. The model achieved an initial AUC of 1, predicting group classifications in the test set. To assess the contribution of each principal component (PC), we permuted them individually and observed the impact on AUC. Only the first principal component (PC1) caused a significant drop in AUC, decreasing it by 0.3. These results indicate that PC1 plays a substantial role in explaining the predictors’ variance and in maintaining the model’s ability to predict class labels accurately. **(B)** shows the components of PC1, with only loading weights greater than 0.2 included in the plot. Thicker lines showing the top 5 weights of the PC1 loadings. Results suggest that FC between thalamo-cortico-cerebellar loop elements with premotor cortex is responsible for the highly accurate predictions of the clinical groups.

To evaluate the contribution of each principal component (PC) to the AUC, we permuted individual components to dissociate them from the group labels. The results showed a 0.3 drop in AUC on the test set when PC1 was excluded from the prediction, while no other principal components caused a significant drop. This indicates that PC1 influences group discrimination the most (See **Figure 6A**). The top five loading weights of PC1 were associated with the ‘after’ condition, including connectivities between Right Insular Cortex – Right Cerebellum 6 (weight (w) = 0.29), Right Thalamus – Right Insular Cortex (w= 0.26), Left Premotor 6d – Right Cerebellum 6 (w=0.26), Left Thalamus – Right Insular Cortex (w=0.25), and Right Thalamus – Left Insular Cortex (w=0.25), as shown by the thick lines in **Figure 6B**. These findings suggest that the thalamo-cortico-cerebellar circuit, also the premotor region, plays a significant role in differentiating schizophrenia from healthy controls. It is important to note that the behavioral data had a low weight on PC1, indicating that it is not a strong predictor for distinguishing between the clinical groups (Mean reaction time = 0.04, Number of correct responses = -0.05). A support vector machine (SVM) model classified patients and controls with high accuracy (AUC = 0.95–1), with task-induced connectivity changes in thalamo-cortico-cerebellar circuits emerging as the strongest predictors of a group membership. Although we conducted this study *a priori*, data-driven analyses confirmed the post-task differences between both groups. For a control analysis, when we included the all ROIs FC in pre- and post-task, AUC was 1 for the test group, highlighting that FC success for the group discrimination not only when group differences are considered as predictors.

## 4 Discussion

In this study, we examined the relationship between implicit learning of schizophrenia patients with neuroplasticity using a reward-enhanced SRTT via rs-fMRI. Our findings showed patients had a lower proportion of correct responses compared to controls, suggesting implicit learning deficits in schizophrenia. The observed baseline hyperconnectivity in cortico-striato-cerebellar regions became even more robust and widespread following task completion in patients. However, increased connectivity was not associated with the patients’ performance. It was further observed that hyperconnectivity after the task was a differentiating feature that could classify individuals into different groups.

A dissociation in behavioral performance was observed in schizophrenia patients. While their reduction in reaction times (RTs) across sequential compared to random blocks was comparable to that of healthy controls—suggesting partially preserved implicit learning—they demonstrated significantly lower accuracy in the sequential blocks. This discrepancy points to potential deficits in motor execution, attentional regulation, or the integration of learned sequences. Additionally, the inclusion of a reward component in the task, together with the group’s relatively low ESRS scores, may have influenced reaction time patterns positively in patients. However, the lack of sequence-specific improvement in the post-test condition, along with a lower proportion of correct responses, further supports the presence of subtle impairments in implicit learning among schizophrenia patients. Our findings are also consistent with the meta-analysis conducted by Siegert et al. (27). In reviewing the studies included in this meta-analysis, for example, Green et al. (29) reported that patients with chronic schizophrenia exhibited significantly lower levels of sequence learning compared to healthy controls. Similarly, Marvel et al. (30) found impaired implicit learning in a group of psychosis, although the inclusion of individuals with schizoaffective disorder was noted as a limitation. To eliminate the effects of medication, Exner et al. (31) investigated drug-naïve first-episode patients and reported that impairments in implicit learning may be related to the nature of the disorder. These findings were supported by Zedkova et al. (32), who also found impaired implicit learning in patients with chronic schizophrenia. Consistent with these findings, our study also showed that patients had lower accuracy compared to controls, suggesting implicit learning deficits.

Our finding of the increased baseline cortico-striato-cerebellar hyperconnectivity in schizophrenia patients has been reported previously from the onset of the disease and has been proposed as a potential soft neurological sign of the disorder (33–37). The observation of hyperconnectivity between the thalamus and cortical regions in the current study supports the idea that the excitatory-inhibitory imbalance between glutamatergic and GABAergic neurons leads to dysfunction in thalamo-cortical neurocircuits (38). Northoff et al. proposed that increased dopaminergic and decreased serotonergic signaling in psychotic disorders, including schizophrenia, may contribute to increased sensorimotor-thalamic functional connectivity (39).

In previous studies, healthy subjects have increased cortico-striato-cerebellar connectivity after SRTT and one study reported healthy controls have more cortico-striato-cerebellar connectivity compared to schizophrenia patients (40). However, we could not observed connectivity changes in the healthy controls. On the other hand, our schizophrenia patients had more widespread increased cortico-striato-cerebellar connectivity after SRTT. The reason for the discrepancy between our and the previous study might be due to the type antipsychotics that the patients were on. In the previous study, those who remained on typical antipsychotics continued to show a lack of procedural learning, while patients who switched to atypical antipsychotics demonstrated significant improvements in procedural learning, accompanied by increased activation in the superior-middle frontal gyrus, anterior cingulate, and striatum (41). In our study, the fact that all patients were using atypical antipsychotics may have contributed to the observed results, potentially due to the relatively weaker nigrostriatal D2 blockade associated with these medications.

Our results show that although cortico-striato-cerebellar hyperconnectivity present in schizophrenia patients, its lack of correlation with behavioral performance supports the dysfunctional brain networks hypothesis in schizophrenia (42). At the cellular level, the disturbances in the integration of electrical stimuli impair the consolidation of incoming information, resulting in excessive information overload in the brain. Consequently, even if connectivity within certain brain networks increases, functional impairments may still occur (43). Additionally, studies using psychedelic-induced psychosis models suggest that abnormal synaptogenesis leads to hyperconnectivity and increased neural entropy (44). However, this hyperconnectivity might not be functionally beneficial as showed in a previous study by Wu et al. (45), the finding that sensorimotor-thalamic hyperconnectivity was associated with poorer task performance further supports the dysfunctional brain networks hypothesis in schizophrenia patients.

In their study, Chang et al. (46) demonstrated the presence of microstructural abnormalities in the cerebellar peduncles of patients with schizophrenia. These abnormalities were specific to the middle and inferior cerebellar peduncles and were characterized by reduced fractional anisotropy and increased radial diffusivity. Notably, these changes were significantly associated with cognitive performance in healthy controls, but not in patients. This suggests that impaired cerebellar white matter integrity may underlie cognitive impairments in schizophrenia. Damage to the cerebellar pathways has been shown to impair efficient information processing, which, in turn, leads to increased functional connectivity within the circuit as a compensatory mechanism to maintain basic task performance.

Recent evidence from several neuropsychiatric disorders suggests that hyperconnectivity within large-scale brain networks may reflect a compensatory response to underlying structural or functional disturbances. For example, patients with mild traumatic brain injury showed increased functional connectivity between the inferior fronto-occipital fasciculus and primary sensorimotor along with cerebellar networks, which was associated with impaired information processing speed, suggesting a maladaptive compensatory mechanism (47). Similarly, patients with generalized tonic-clonic seizures showed increased variability in functional connectivity, particularly in the default mode, attention and cognitive control networks, reflecting a dynamic reorganization that may be related to both epileptic activity and cognitive dysfunction (48). Consistent with these findings, our study in schizophrenia revealed hyperconnectivity within the cortico-striato-cerebellar circuit, which we interpret as a compensatory adaptation to widespread disruptions in brain function. Taken together, these findings support the notion that increased network connectivity may serve as a general compensatory mechanism in neuropsychiatric disorders, albeit potentially associated with inefficient cognitive processing.

In this study, we employed a reward-related SRTT, which is expected to modulate neural activity in emotion-related regions. Our findings revealed reduced connectivity between the insula and the posterior cingulate cortex (PCC), a key region of the default mode network (DMN). This result aligns with Ebisch et al. (49), who reported similar disruptions in first-episode schizophrenia patients, suggesting that schizophrenia may impair the insula’s role in emotional processing (50).

Additionally, we observed increased insula-thalamus connectivity following the task, consistent with previous findings that connectivity in this pathway strengthens with feedback (51). This suggests that schizophrenia patients remain responsive to feedback mechanisms. Furthermore, we found enhanced connectivity between the insula, a core component of the salience network, and the cerebellum in schizophrenia. This may reflect a compensatory mechanism for executive dysfunction, potentially engaging the reward system.

While Chrobak et al. (52) reported no relationship between the sensorimotor network and SRTT performance, our findings suggest that using a reward-enhanced SRTT may have contributed to increased connectivity between the premotor cortex (PMC), a key sensorimotor region, and the nucleus accumbens. This indicates that despite neuroplasticity deficits, schizophrenia patients may still process rewards, albeit through alternative neural pathways.

Finally, contrary to the meta-analysis by Radua et al. (53), we found increased connectivity between the nucleus accumbens and both the thalamus and PMC. This discrepancy may stem from the fact that all participants in our study were treated with atypical antipsychotics, which are known to normalize striatal activity. Overall, our findings suggest that implicit learning deficits in schizophrenia are not solely attributable to motivational factors or negative symptoms, as often debated, but are likely rooted in fundamental neural dysfunctions, including impaired neuroplasticity.

Our findings demonstrate that SRTT-induced changes can predict clinical groups with high accuracy (AUC: 0.95–1). Key predictors included the PMC, bilateral insula, thalamus, and cerebellum (**Figure 6**). To assess the necessity of neuroimaging, behavioral metrics (reaction times, correct response rates) were included as predictors. However, their weak contributions to PC1, with significant weights (>0.2) only in the post-task fMRI condition, highlight the primacy of neural changes in classification. This suggests that fMRI-based functional connectivity provides deeper insights into neuroplasticity than behavioral measures alone, particularly in task-induced resting-state alterations. Further studies are needed to explore these interactions in greater detail and clarify their role in reward processing and its dysregulation in schizophrenia.

## 5 Limitations

This study has limitations, both in sample size and study design. One limitation is the inability to perform fMRI during the task. This was mainly due to the task’s total duration of approximately 75 minutes, making it challenging for patients to minimize their head movements. Relatively small sample size, the exclusion of left-handed participants, and the cross-sectional design decrease the generalizability of the findings. Additionally, the small sample size may contribute to an overestimation of the SVM results; however, the baseline AUC values appear sufficiently robust to support the findings. Similarly, external validation is needed further to support the effectiveness of SRTT in group distinction. Future research should use longitudinal designs and multimodal imaging to better understand neuroplasticity dynamics and implicit learning over time. Additionally, studying unmedicated patients or those at different illness stages may provide further insights.

## 6 Conclusion

This study provides evidence of altered neuroplasticity in schizophrenia, characterized by hyperconnectivity within cortico-striato-cerebellar circuits during implicit learning. While this adaptation may partly compensate for neuroplasticity deficits, it appears insufficient to fully support task-specific learning. These findings emphasize the importance of targeting neuroplasticity in the development of interventions, such as non-invasive brain stimulation techniques (TMS, tDCS) or pharmacological modulation of glutamate and dopamine systems, to help improve cognitive impairments in schizophrenia. These findings contribute to a growing understanding of the neural basis of implicit learning deficits in this population.

## Data Availability

The raw data supporting the conclusions of this article will be made available by the authors, without undue reservation.
