# Supplementary material for: The effect of implicit learning on functional connectivity in schizophrenia
Source: Front Psychiatry. 2025 Jun 17;16:1600449. doi: 10.3389/fpsyt.2025.1600449 (PMC12209253; doi:10.3389/fpsyt.2025.1600449)
Supplement: Supplementary file 1 [file SupplementaryFile1.docx]

Supplementary Material

2.4. MRI acquisition

MRI scans data were collected at the Department of Radiology, Ege University using a 3.0 Tesla MRI scanner with a 12-channel head coil (Siemens Magnetom Verio, Numaris/4, Syngo MR B17, Erlangen, Germany) was used for imaging.

The MRI protocol included axial sections with TRA T2-weighted sequences obtained using the BLADE technique (TR/TE: 2320/117 ms, slice thickness: 5 mm, number of slices: 20, inter-slice gap: 2 mm, voxel size: 0.7x0.7x5 mm, FOV: 220, Nex: 1, GRAPPA factor: 2); coronal sections with T2 FLAIR COR sequences (TR/TE/TI: 9000/85/2500 ms, slice thickness: 4 mm, number of slices: 38, no inter-slice gap matrix: 192x256, voxel size: 1.1x0.9x4 mm, FOV: 220, Nex: 1, GRAPPA factor: 2); and sagittal sections with T1-weighted 3D MP-RAGE sequences (TR/TE/TI: 1600/2.21/900 ms, FA: 9, slice thickness: 1 mm, number of slices: 160, no inter-slice gap matrix: 246x256, voxel size: 1x1x1 mm, FOV: 256, Nex: 1, GRAPPA factor: 2). T2-weighted Echo-planar imaging (EPI) scans were acquired for the task paradigm. EPI parameters were like following; 42 × 3mm slices, interleaved from bottom to top (interslice gap: 1mm, TE: 30ms, TR: 3000ms, flip angle: 90°, FOV: 192×192mm, in-plane matrix resolution: 64×64, 1056 dynamic scans with 2-s duration).

2.4.1.Resting State fMRI Preprocessing

Functional and anatomical data were preprocessed using a modular preprocessing pipeline including realignment with correction of susceptibility distortion interactions, slice timing correction, outlier detection, direct segmentation and MNI-space normalization, and smoothing. Functional data were realigned using SPM realign & unwarp procedure, where all scans were coregistered to a reference image (first scan of the first session) using a least squares approach and a 6 parameter (rigid body) transformation and resampled using b-spline interpolation to correct for motion and magnetic susceptibility interactions. Temporal misalignment between different slices of the functional data (acquired in interleaved Siemens order) was corrected following SPM slice-timing correction (STC) procedure, using sinc temporal interpolation to resample each slice BOLD time-series to a common mid-acquisition time. Potential outlier scans were identified using ART as acquisitions with framewise displacement above 0.9 mm or global BOLD signal changes above 5 standard deviations, and a reference BOLD image was computed for each subject by averaging all scans excluding outliers. Functional and anatomical data were normalized into standard MNI space, segmented into grey matter, white matter, and CSF tissue classes, and resampled to 2 mm isotropic voxels following a direct normalization procedure using SPM unified segmentation and normalization algorithm with the default IXI-549 tissue probability map template. Finally, functional data were smoothed using spatial convolution with a Gaussian kernel of 8 mm full-width half maximum (FWHM). In addition, functional data were denoised using a standard denoising pipeline including the regression of potential confounding effects characterized by white matter time-series (5 CompCor noise components), CSF time-series (5 CompCor noise components), motion parameters (6 factors), outlier scans (below 59 factors), session and task effects and their first order derivatives (4 factors), and linear trends (2 factors) within each functional run, followed by bandpass frequency filtering of the BOLD time-series between 0.008 Hz and 0.09 Hz. CompCor noise components within white matter and CSF were estimated by computing the average BOLD signal as well as the largest principal components orthogonal to the BOLD average, motion parameters, and outlier scans within each subject's eroded segmentation masks. From the number of noise terms included in this denoising strategy, the effective degrees of freedom of the BOLD signal after denoising were estimated to range from 95.4 to 135.8 (average 130.5) across all subjects.

**Imaging results:**

**Supplementary Table 1. MNI Coordinates of Selected ROI Regions**

| **ROI** | **MNI Coordinates** | | |
| --- | --- | --- | --- |
|  | X | Y | Z |
| Cerebelum_10_R | 26 | -34.1 | -41.5 |
| Cerebelum_10_L | -22.7 | -34.1 | -41.5 |
| Cerebelum_9_R | 9.8 | -49.6 | -46.7 |
| Cerebelum_9_L | -10.9 | -48.8 | -46 |
| Cerebelum_8_R | 25.3 | -56.2 | -49.6 |
| Cerebelum_8_L | -25.6 | -54.7 | -47.4 |
| Cerebelum_7b_R | 34.9 | -63.6 | -47.4 |
| Cerebelum_7b_L | -33 | -59.9 | -43 |
| Cerebelum_6_R | 24.5 | -58.4 | -23.8 |
| Cerebelum_6_L | -23.4 | -59.1 | -22.4 |
| Cerebelum_4_5_R | 17.2 | -42.9 | -17.9 |
| Cerebelum_4_5_L | -15.3 | -43.7 | -17.2 |
| Cerebelum_3_R | 12 | -34.8 | -19.4 |
| Cerebelum_3_L | -8.7 | -37 | -18.7 |
| Cerebelum_Crus2_R | 31.9 | -68.7 | -40.1 |
| Cerebelum_Crus2_L | -28.6 | -73.2 | -38.6 |
| Cerebelum_Crus1_R | 37.8 | -67.3 | -29.7 |
| Cerebelum_Crus1_L | -35.9 | -66.5 | -29 |
| Thalamus_R | 12 | -17.8 | 7.9 |
| Thalamus_L | -11.6 | -17.8 | 7.9 |
| Pallidum_R | 20.1 | -0.1 | -0.2 |
| Pallidum_L | -19 | -0.1 | -0.2 |
| Putamen_R | 26.7 | 5 | 2.7 |
| Putamen_L | -24.9 | 3.5 | 2.7 |
| Caudate_R | 14.2 | 12.4 | 9.4 |
| Caudate_L | -12.3 | 10.9 | 9.4 |
| Insula_R | 37.8 | 6.5 | 2 |
| Insula_L | -35.9 | 6.5 | 3.5 |
| Accumbens_R | 12.7 | 9.4 | -11.3 |
| Accumbens_L | -13.1 | 7.2 | -12 |
| Posterior Cingulate_R | 6.8 | -42.2 | 21.9 |
| Posterior Cingulate_L | -5.7 | -42.9 | 24.8 |
| 6a_premotor_L | -25.6 | -1.6 | 54.3 |
| 6d_premotor_L | -35.2 | -12.7 | 66.9 |
| 6r_premotor_L | -52.2 | 7.2 | 17.5 |
| FEF_premotor_L | -41.1 | -4.6 | 52.9 |
| PEF_premotor_L | -47.7 | 0.6 | 41.1 |
